# Reduction by air purifier of particulate concentration during orthodontic procedures: a pilot study

**DOI:** 10.1186/s12903-024-03956-w

**Published:** 2024-02-07

**Authors:** Inmaculada Martín-Quintero, Alberto Cervera-Sabater, Jorge Cortés-Bretón Brinkmann, Juan Manuel Aragoneses-Lamas, Javier Flores-Fraile, Juan Santos-Marino

**Affiliations:** 1 Department of Surgery, Faculty of Medicine, University of Salamanca, Salamanca, 37007 Spain; 2 Faculty of Dentistry, Universidad Alfonso X El Sabio, Villanueva de la Cañada, 28961 Spain; 3 Department of Dental Clinical Specialties, Faculty of Dentistry, Complutense University of Madrid, Madrid, 28049 Spain

**Keywords:** Bioaerosols, COVID-19, Dentistry, Orthodontics, SARS-CoV-2, air filters, Air Purifier

## Abstract

**Background:**

The SARS-CoV-2 pandemic has raised awareness of the importance of air quality. This pilot study arose from the need to reduce the concentration of particulate matter in the dental office during orthodontic procedures. To evaluate the efficacy of using an air purifier during orthodontic care in the dental office to reduce the concentration of ambient particulate matter.

**Results:**

Significant reductions in particle numbers were obtained for all particle sizes except the largest particles counted (10 μm) through use of the air filter. A marked association between higher humidity levels and higher particle counts was also observed.

**Conclusions:**

Using an air purifier during dental care achieves a significant reduction in the concentration of ambient particles in the dental office. There is a correlation between higher relative humidity and higher particle concentration. The probability of obtaining a maximum particulate concentration level of 0.3 and 0.5 μm is 1000 times lower when using an air purifier.

## Background

In recent years, air quality has become an increasingly important concern and interest in the topic has grown exponentially since the outbreak of the SARS-CoV-2 pandemic [1]. The effects of environmental pollution on the quality of life and health of the population are well known [1, 2]. 

Strong short-term associations between particulate matter less than 10 μm (PM_10_) and less than 2.5 μm (PM_2.5_) and deaths from cardiovascular and/or respiratory causes have been observed in more than 600 cities worldwide, reinforcing the evidence for a correlation between particulate matter concentration and mortality [2–4].

Over the last century, the environment has become a source of air pollutants that include millions of nanoparticles that carry pathogens [2–4]. The proportion of nanoparticles in the surrounding environment influences our state of health significantly, as nanoparticles can enter our bodies through the respiratory tract, reach the bloodstream, and then the functional tissues [5]. Moreover, even a slight increase in exposure to higher concentrations of small particulate matter in the long term has been found to lead to a major increase in the COVID-19 mortality rate [2–5]. 

This highlights the importance of implementing air pollution regulations to protect people’s health both during the COVID-19 crisis and thereafter [4, 5].

The SARS-CoV-2 virus is very stable at low temperatures but very sensitive to heat. The virus’s dominant transmission route is respiratory [6]. Scientific evidence indicates that viruses that are found in aerosols are more transmissible under certain circumstances including indoor medical settings and poorly ventilated interiors [6–9]. However, indoor air quality (in workplaces, homes…) is not paid the attention it deserves. The exponential increase in respiratory diseases in Western society over the last forty years cannot be explained without placing indoor air quality high on the list of causes. Occupational exposure to pollutants is not limited to industrial or similar environments but has also been observed in many workplaces such as offices and other interior spaces [1, 5].

The pandemic has prompted a search for ways of ensuring a safer environment in dental offices. These include the use of air purifiers and early results indicate that they have a positive effect on indoor air quality [10]. Recent studies indicate that the use of HEPA filters significantly increase aerosol removal and decrease aerosol accumulation, especially in rooms with low ventilation rates [11, 12].

In the future, we are likely to see the emergence of strong evidence for the association between air pollution and COVID-19. Air quality may prove another tool at our disposal for assessing COVID-19 infectivity and patterns of future infection [9–11]. This could allow policy makers to establish proactive strategies for dealing with future pandemics, prioritizing regions with high air pollution. In this context, long-term strategies for air quality protection and enforcement are of great importance [7, 13–15]. At the same time, more studies are needed to validate the aerosol transmission of SARS-CoV-2 that would help our understanding of its infectivity and spreadability. This could help prevent outbreaks in indoor settings such as hospitals and other facilities by ensuring well-designed and efficiently ventilated healthcare environments [13, 16].

Recently, several studies have been conducted on particle concentration in dental offices, especially during everyday orthodontic practice. These have highlighted the importance of quantifying oscillations in particle concentrations. In addition, they have identified a number of variables that can be controlled to achieve safer concentration levels [17].

The aim of this study was to determine whether the use of air purifiers in the dental office during orthodontic procedures reduces the concentration of particulate matter in the environment significantly, and therefore the associated risks to patients and staff. If this hypothesis can be confirmed, a preliminary protocol could be established to reduce the number of indoor airborne particles in the dental office.

## Methods

This experimental study was conducted during a series of 58 consecutive appointments scheduled for patients attending the clinic attached to the Master’s Program in Orthodontics at the Alfonso X El Sabio University (Madrid, Spain), between April and May 2022.

Patients attending the clinic gave their consent for the study to be conducted during their appointments. The study design followed guidelines established in the Helsinki Declaration for medical research and was approved by the Bioethical Research Committee of the Faculty of Sciences of the University Alfonso X El Sabio (2022_1/118).

The study was carried out in a single room of limited size located at the center of the university dental specialties clinic. This space is open on one side and has a forced air renewal air conditioning system. Each dental cabinet is equipped with a ventilation unit. This unit filters the air that is introduced into the centre, providing automated scheduling for the air-conditioning system.

**Fig. 1:**
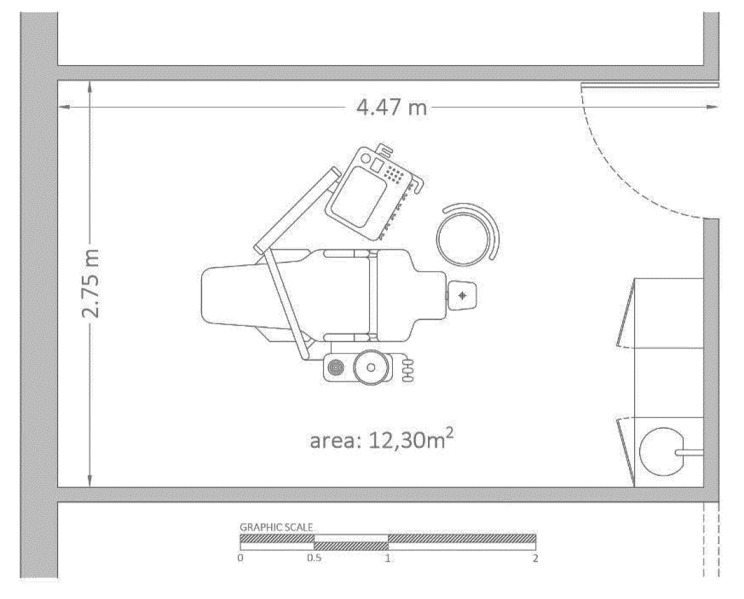
Floorplan of the operation room

The dental cabinet is 2 m in height and has the dimensions specified in the floor plan in Fig. 1.

The procedures carried out during the patients’ appointments consisted of revisions of fixed orthodontic appliances including changing archwires, cementing detached brackets, placing accessory attachments for biomechanics, placing ligatures, and taking impressions. In every case, a water-air syringe was used at some point, and in certain instances, a rotary instrument. High-speed rotary instruments were not used in any case; low-speed rotary instruments were only used when the procedures required it.

Patients were treated by a single clinician in order to avoid biases arising from a variety of uncalibrated operators. Airborne particle measurement was performed at a height of 150 cm and a separation of 50 cm from the patient’s head.

Measurement consisted of a continuous recording of the particles generated every minute during the session. The recording with the highest number of 0.3 μm particles was selected for later comparison. At each measurement, the number of particles (0.3 μm, 0.5 μm, 1 μm, 2.5 μm, 5 and 10 μm) contained in 1 L of air every minute, temperature (Tª), and relative humidity (RH) were recorded. Measurements were taken with the TROTEC® cleanroom particle counter model 220 (ISO 21501-4) calibrated by the manufacturer, adjusted to measure the particles contained in 1 L of air. The device is intended for measuring scattered light. To confirm the data obtained, the procedures were recorded on video with a count superimposed on the image (using the device’s recording setting).

The records collected were divided into two groups. The control group consisted of records taken during the orthodontic session with the filter present but not turned on. The study group comprised data collected during sessions with the air filter in operation.

The air filter used was the BIOW100 with multistep HEPA filters in automatic operation mode. The decibel range of this device is 20.4–41.9dB. The operator and the patient were unaware beforehand whether the filter was switched on or not. Even though the device is quite noiseless and operated during the care activity, making it practically imperceptible, it cannot be confirmed whether or not they were aware of the device being on or off.

In order to minimize the influence of variations in environmental conditions on the data recorded, the day’s work was randomly divided between study sessions and control sessions with the air filter operating or switched off. Neither the clinician nor the patient was aware of whether the filter was operating or not.

Statistical analysis was performed with: the SPSS V25, IBM Corp. 2017 statistical software package; and IBM SPSS Statistics for Windows, Version 25.0. Armonk, NY: IBM Corp. The Kolmorogov-Smirnov test was performed to test variables normality and determined parametric or non-parametric distribution. For comparing the two groups, Student’s test was performed for parametric quantitative variables and Mann-Whitney U test for quantitative non-parametric variables, Spearman and Pearson’s correlations were applied to analyze relationships between variables.

## Results

The correlation between the use of filters (independent variable) and the number of 0.3 μm particles (dependent variable), with a regression coefficient of -0.284, shows a negative or inverse trend. This means that with less filter usage, the number of 0.3 μm particles increases, regression coefficient *r*= -0.284 (*p* = 0.031), statistically significant.

The correlation between the use of filters (independent variable) and the number of 0.5 μm particles (dependent variable), with a regression coefficient of -0.296, also shows a negative or inverse trend. This means that with less filter usage, the number of 0.5 μm particles increases, regression coefficient *r*= -0.296 (*p* = 0.024), statistically significant.

The correlation between the use of filters (independent variable) and the number of 1 μm particles (dependent variable), with a regression coefficient of -0.292, shows a negative or inverse trend. This means that with less filter usage, the number of 1 μm particles increases, regression coefficient *r*= -0.292 (*p* = 0.026), statistically significant.

The correlation between the use of filters (independent variable) and the number of 2.5 μm particles (dependent variable), with a regression coefficient of -0.262, shows a negative or inverse trend. This means that with less filter usage, the number of 2.5 μm particles increases, regression coefficient *r*= -0.262 (*p* = 0.047), statistically significant.

The correlation between the use of filters (independent variable) and the number of 5 μm, 10 μm particles and humidity (dependent variables), with a regression coefficient of -0.211, -0.174, -0.174, shows a negative or inverse trend. This means that with less filter usage, the number of 5 μm, 10 μm particles and increased humidity increase, regression coefficient *r*=-0.211, -0.174, -0.174 (p = > 0.050), statistically not significant.

The Table 1 shows the correlation results obtained between the number of particles, humidity, and temperature.

**Table 1:** Table of correlation results between particle number, humidity, temperature, and filter use

Variables	Beta Unstandardized Coefficients	Beta Standardized Beta Coefficients	*p*-value	95.0% C.I lower	95.0% C.I upper
0.3 μm vs. Filter use	-3107,077	-0,284	0,031	-5915,885	-298,269
0.5 μm vs. Filter use	-1770,426	-0,296	0,024	-3299,086	-241,766
1.0 μm vs. Filter use	-523,492	-0,292	0,026	-983,115	-63,868
2.5 μm vs. Filter use	-118,899	-0,262	0,047	-235,977	-1,821
5 μm vs. Filter use	-4,256	-0,211	0,112	-9,533	1,021
10 μm vs. Filter use	-5,897	-0,174	0,192	-14,846	3,051
Temperature vs. Filter use	,251	0,129	0,334	-0,264	0,765
Humidity vs. Filter use	-2,194	-0,174	0,190	-5,509	1,121

Statistical tests: Student’s T-test and Mann-Whitney U test. * Significant differences found, statistical significance was considered at *p* < 0.05

During the recording sessions, mean temperature was 24.89 ºC, SD ± 0.97, median 24.93, range 22.66–27.10. Table 2 shows the temperatures recorded in both the study group and control group. There was no significant difference between the groups (Mann-Whitney U test, *p* = 0.260, df = 1).

**Table 2:** Temperatures recorded in study group and control group

Group	Mean	SD	Median	Range	*p*-value
Filter	25.01	1.03	25.00	22.66–26.73	0.260
Control	24.76	0.92	24.86	23.22–27.10	0.260

Temperature in (degrees Celsius Cº). Statistical test: Mann-Whitney U test

Mean relative humidity was 34.37%, SD ± 6.34, median 33.24, range 22.00–46.00. Table 3 shows relative humidity recorded in the study group and the control group. No significant difference was found between the groups (Student’s T-test, *p* = 0.191, df = 56).

**Table 3:** Relative humidity in study and control group

Group	Mean	SD	Median	Range	*p*-value
Filter	33.27	5.27	33.00	22.50-45.45	0.191
Control	35.47	7.18	34.37	22.00–46.00	0.191

Statistical test: Student’s T-test

Comparison of the numbers of particles found in the control group and the study group exhibited statistically significant differences. Data analysis found a correlation between levels of relative humidity and the concentration of particles of different sizes (Table 4). It was also observed that the higher the relative humidity in the clinic, the higher the number of particles present.

**Table 4:** Correlation between humidity levels and particle size

Particle Size (µm)	Trend	Correlation Coefficient	*p*-value
0.3	Positiva	0.629	0.0001
0.5	Positiva	0.584	0.00001
1.0	Positiva	0.553	0.00007
2.5	Positiva	0.454	0.0003
5.0	Positiva	0.468	0.0002
10.0	Positiva	0.303	0.021

**Fig. 2:**
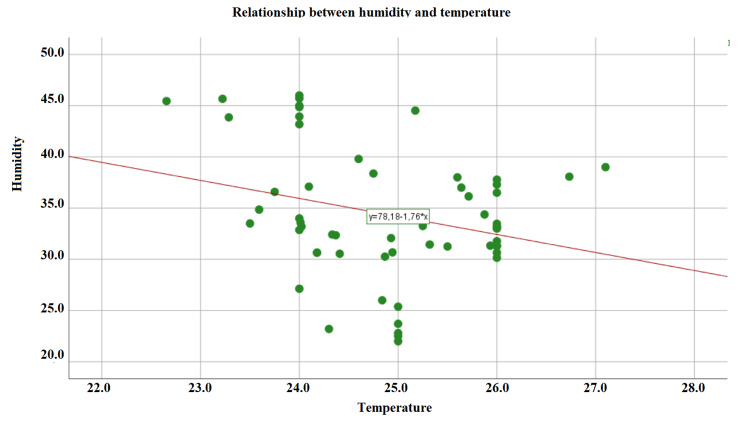
Relationship between relative humidity and temperature

Figure 2 shows the correlation between humidity levels and temperature. The trend was negative. The lower the temperature, the higher the humidity level. Correlation coefficient − 0.271, *p* = 0.039.

Lastly, multivariate analysis, logistic regression findings:

A logistic regression analysis is conducted to determine if there is a relationship between the variables and the usage of filters in the overall sample. In Table 5, significant variables are shown, being predictors (the presence of these variables increases or decreases the probability of filter usage), Omnibus test (*p* = 0.023), R2 = 5.20, the logistic regression model predicts that 68.96% of filter usage and 51.72% without filter usage, making the logistic regression model suitable for analysis.

**Table 5:** Correlation analysis of filter usage based on particle size

		No	Yes	%	Chi-square	Nagelkerke R Square	Omnibus test *p*-value
Filter	No	15	14	51,72	5.203	0.114	0.023
	Yes	9	20	68,96			
	Overall Percentage			60,34			

Logistic regression is carried out to demonstrate the relationship between the variables and the probability of presence and usage of the filter.

**Table 6:** Shows the odd ratio (OR) of the studied variables at the 95% confidence interval

Variables	B coefficient beta	Wald Valid index	*p*-value: statistical significance	OR: Odd ratio	95.0% C.I lower	95.0% C.I upper
Particles 0.3 μm	0,0001	0,088	0,767	1,000	0,999	1,001
Particles 0.5 μm	0,001	0,107	0,743	1,001	0,997	1,004
Particles 1.0 μm	-0,001	4,462	0,035	0,999	0,999	1,000
Particles 2.5 μm	0,001	0,007	0,933	1,001	0,970	1,034
Particles 5.0 μm	0,091	0,943	0,332	1,095	0,911	1,316
Particles 10 μm	-0,004	0,002	0,965	0,996	0,843	1,177
Temperature	0,182	0,235	0,628	1,200	0,575	2,504
Humidity	0,012	0,037	0,848	1,012	0,898	1,139

In Table 6 the OR, *p*-value, and 95% confidence interval of the independent variables that contribute to the probability of using the filter in the overall sample can be observed.

The following variables are associated with a greater probability of filter usage:

- A smaller number of 1.0 μm particles results in a 0.99 higher probability of filter usage (*p* = 0.035).
- A smaller number of 10 μm particles results in a 0.99 higher probability of filter usage (*p* = 0.96).

The following variables are associated with a lower probability of filter usage:

- A larger number of 0.3, 0.5, 2.5, and 5.0 μm particles results in a 1.0 lower probability of filter usage (p > = 0.050).
- Higher humidity and temperature result in a 1.2 lower probability of using the filter (p > = 0.050).

**Fig. 3:**
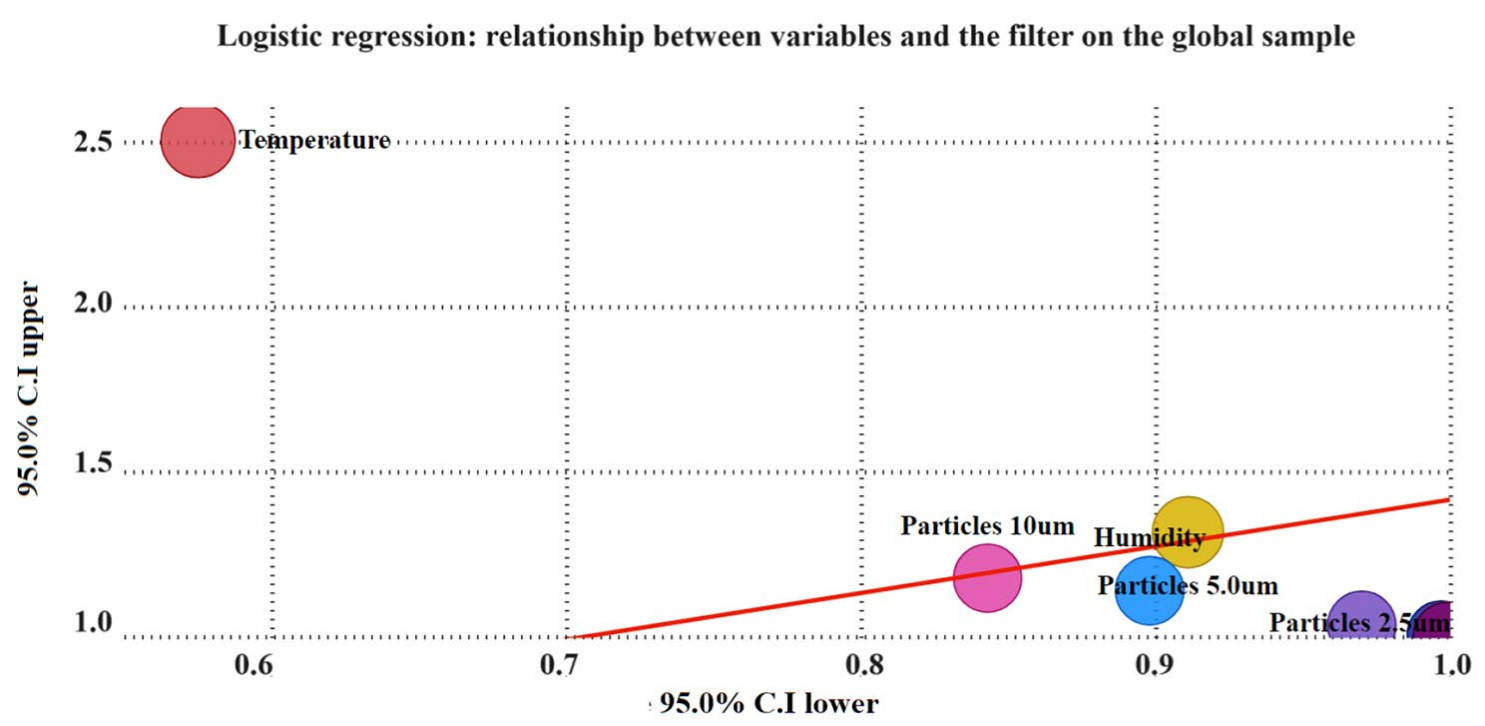
Logistic regression: relationship between variables and the filter on the global sample

Subsequently, Fig. 3 depicts the logistic regression representing the correlation between the variables and the application of the filter.

## Discussion

The COVID-19 pandemic has brought attention to the importance of indoor air quality in healthcare settings, including dental clinics. Airborne diseases can pose a risk to both patients and healthcare professionals, making it crucial to explore measures to reduce indoor pollution. This study aimed to evaluate the impact of air filters and extraoral sweeping devices (EOS) on particle concentrations during daily orthodontic sessions. Understanding the relationship between temperature, humidity and particle concentration is vital, as it can influence the quality of the indoor environment and the transmission of pathogens [4, 5, 13, 16].

The present study measured the amount of particulate matter in the dental office during daily orthodontic sessions with and without the use of an air filter. The data collected in the two groups (study group with filter and control group without filter) were recorded on the same day, which avoided major variations in temperature and relative humidity. The stability of these variables was an important factor for comparing data between the two groups, as previous studies have shown that temperature affects humidity and humidity influences the concentration of larger particles [17–19]. The presence of larger particles diminishes indoor environmental quality, increasing virus transmissibility and virus survival rates [18].

Analyzing the present data confirmed the relationship between higher temperature levels and lower relative humidity levels. It was observed that as relative humidity decreased, there was a corresponding decrease in the concentration of particles of the different sizes counted [17, 18].

The systematic review of 517 articles by Mecenas P et al. found that COVID-19 transmission was favoured in warmer and more humid climates. However, the evidence found was of low quality due to the limited consistency of the included studies [19]. Data were collected from a study group using an air filter and a control group without a filter, both on the same day to minimize temperature and humidity variations. Particulate concentrations were measured to assess the effectiveness of the air filter. As mentioned above the review by Mecenas et al. suggested a possible relationship between COVID-19 transmission and temperature and humidity levels, but more research is needed due to the heterogeneity of existing studies [19].

Since the SARS-CoV-2 pandemic, air quality – especially indoor air quality – has become an even important matter. While regulating environmental conditions influences many aspects of human health, COVID-19 has elevated this concern significantly among both healthcare professionals and patients. In the field of dentistry, COVID-19 disease and its transmission routes have reopened the debate about risks to dentists in various specializations. Orthodontics has always been considered one of the less risky practices because it does not involve much exposure to patients’ blood. However, as the pandemic has again reminded us, airborne diseases also present a risk to the general population, to patients, and to all healthcare professionals, including dentists [20, 21].

Dental instruments generate aerosols that may pose risks to operators due to the transmission of pathogens, such as the SARS-CoV-2 virus [22]. Due to the SARS-CoV-2 outbreak, aerosol control in the dental office has become a critical safety issue in dentistry. The use of extraoral sweeping devices (EOSs) is one of several approaches to aerosol reduction during treatment in dentistry. Nevertheless, the application and efficacy of EOS in the dental environment is still debated in the literature and questions still remain open about their appropriate use. Therefore, further research in this area is essential to work towards a safer dental practice [23–25]. 

Nonetheless, the study by Yang M et al. concluded that the increase in the level of aerosols smaller than 10 μm was minimal during dental procedures when using the saliva ejector and high-speed suction [25]. 

In this context, the use of air filters may be a viable means of reducing the airborne transmission of pathogens [21, 26]. Duill et al. investigated the impact of air purifiers with HEPA filters on a school environment, obtaining positive reductions in bioaerosols [26]. At the same time, several studies have concluded that many air filters fail to remove respiratory aerosols effectively. But indoor air purifiers with High Efficiency Particulate Air (HEPA) filters may be used effectively to filter polluted indoor air providing the HEPA filters are replaced regularly [27].

Experimental studies in the literature agree that HEPA purifiers offer great potential to decontaminate, eliminate the airborne pathogen load, and improve indoor air quality. Normally, rooms should be left closed and unused for a short period after aerosol-generating procedures, but with a HEPA purifier this time can be reduced [21, 28].

Lednicky et al. assessed the capability of HEPA filters to efficiently capture and remove SARS-CoV-2 viral particles in a simulated laboratory environment. The results indicated that HEPA filters achieved effective removal of viral particles, supporting their role in preventing virus transmission in indoor spaces [28]. 

The study by Allen et al. evaluated the effectiveness of HEPA filters in reducing fine particulate matter (PM2.5) and volatile organic compounds (VOC) in residential homes. The results showed a significant decrease in PM2.5 and VOC levels after the installation of HEPA filters in heating and air conditioning (HVAC) systems. This reduction in pollutant levels can have a positive impact on the respiratory health of the occupants of these spaces [29]. 

The systematic review by Liu et al. demonstrated portable HEPA purifiers to be effective in the removal of SARS-CoV-2 viral particles in indoor air. However, their use is recommended in combination with other preventive measures to maximize effectiveness in reducing the risk of COVID-19 transmission in enclosed spaces [30]. 

Tzoutzas et al. concluded that good ventilation and the use of air purifiers can improve air quality, observing a reduction in concentration levels of 2.5 μm particulate matter in a university dental clinic during standard dental care activities [31].

Although the epidemiological characteristics of SARS-CoV-2 have not been fully clarified, there are indications that it spreads more during winter, pointing to the influence of temperature and humidity. Elsaid et al. recommend a room temperature of 25–27 °C and replacing HEPA filters with nano-fiber air filters or electrostatic filters in central air conditioning systems [32].

The afore mentioned is consistent with the findings of the study by Yang M [25], Fenelly et al. which concluded that properly placed high-volume evacuation and local exhaust ventilation is effective in avoiding the airborne spread and permanence of inhalable particles from dental procedures [33]. 

Dental offices are potential sources of aerosols carrying pathogens such as SARS-CoV-2 virus. Aerosol control measures are crucial to maintain the safety of healthcare personnel and patients. The use of EOS and high-speed suctioning during dental procedures has been shown to minimize the spread of aerosols. However, the application and efficacy of EOS in dental settings is still under debate in the literature [10, 17, 18, 34].

Further studies are needed to establish effective measures and protocols for reducing the concentration of particulate matter in dental offices [21, 24]. If we are to improve the environmental quality in dental offices, in addition to testing air filters like the one evaluated here, research should also focus on the impact of the continuous use of dental suction systems and the use of dehumidifiers.

## Conclusions

In this pilot study, significant correlations were found between the use of the filter (BIOW100 air purifier with multistage HEPA filters) in daily orthodontic consultations and the number of particles of different sizes. The results suggest that less frequent use of the filter corresponds to an increase in these particles in the air. These results indicate that smaller particle counts and lower humidity and temperature correlate positively with filter usage.

Further studies are needed that aim to improve air quality in dental offices and make them a healthier environment for both staff and patients.

## Data Availability

The datasets used and/or analysed during the current study available from the corresponding author on reasonable request.

## Declarations

### Limitations of the study

In this study we encountered the limitations of the sample size and those associated with the environmental conditions of air renewal or movement within the room.

Due to the design of the study, we did not discriminate between the different procedures included in orthodontic revision appointments.

No previous studies on environmental particles in an orthodontic practice prior to or during the current pandemic have been found in the literature.

Laser devices, such as the one used, show greater measurement inaccuracies when measuring smaller particles. Consequently, we can expect larger errors in the measurements of these particles.
